# Prognostic relevance of mutations and copy number alterations assessed with targeted next generation sequencing in IDH mutant grade II glioma

**DOI:** 10.1007/s11060-018-2867-8

**Published:** 2018-04-16

**Authors:** Maarten M. J. Wijnenga, Pim J. French, Hendrikus J. Dubbink, Winand N. M. Dinjens, Peggy N. Atmodimedjo, Johan M. Kros, Ruth Fleischeuer, Clemens M. F. Dirven, Arnaud J. P. E. Vincent, Martin J. van den Bent

**Affiliations:** 1 Department of Neurology, Brain Tumor Center at Erasmus MC Cancer Institute, PO Box 5201, 3008AE Rotterdam, The Netherlands; 2 Department of Pathology, Brain Tumor Center at Erasmus MC Cancer Institute, Rotterdam, The Netherlands; 3 Department of Pathology, St Elisabeth Hospital, Tilburg, The Netherlands; 4 Department of Neurosurgery, Brain Tumor Center at Erasmus MC Cancer Institute, Rotterdam, The Netherlands

**Keywords:** Glioma, Low-grade, Molecular markers, IDH, 9p21.3, 7q

## Abstract

**Background:**

At current prognostication of low grade glioma remains suboptimal and might be improved with additional markers. These may guide treatment decisions, in particular on early adjuvant therapy versus wait and see after surgery.

**Methods:**

We used a targeted Next-Generation Sequencing panel to assess mutational and copy number status of selected genes and chromosomes in a consecutive series of adult grade II supratentorial glioma, and assessed the impact of molecular markers of interest on overall survival.

**Results:**

207 *IDH* mutated grade II glioma samples were analyzed with a median follow-up of 6.9 years. Loss of region 9p21.3 did not show a correlation with outcome in *IDH* mutated 1p/19q-codeleted oligodendroglioma or *IDH* mutated astrocytoma. We found a significant shorter overall survival with univariable analysis in *IDH* mutated astrocytoma patients with trisomy of chromosome 7 (Log rank P = 0.044) and in *IDH* mutated 1p/19q-codeleted oligodendroglioma patients with a *PTEN* mutation (Log rank P = 0.033). We could not validate these findings in multivariate analysis or in the TCGA dataset.

**Conclusions:**

Loss of 9p21.3 is not associated with outcome in a molecularly defined cohort of grade II glioma and therefore it remains unclear if loss of 9p21.3 can be used as additional marker of anaplasia or to guide treatment decisions. Trisomy of chromosome 7 in *IDH* mutated astrocytoma and *PTEN* mutations in *IDH* mutated oligodendroglioma are potential markers of poor prognosis, but require confirmation in larger series.

**Electronic supplementary material:**

The online version of this article (10.1007/s11060-018-2867-8) contains supplementary material, which is available to authorized users.

## Introduction

In 2016 the World Health Organization Classification of Tumors of the Central Nervous System (WHO) was updated, resulting in a major change in the classification of diffuse gliomas [1]. The 2016 WHO classification provides an integrated diagnosis of glioma by combining histopathological with genotypic features. Moreover, in the case of discrepancy between genotypic and histopathological features, the genotypic features are leading in classifying a glioma subtype. As a result the 2016 CNS WHO markedly improved the objectivity of classification and prognosis estimation as compared to the previous version [1–3].

Three clinically relevant subgroups of diffuse low-grade (grade II) gliomas are identified and recognized by the WHO 2016 classification based on two molecular markers: (1) oligodendroglioma, *IDH* mutant and 1p/19q-codeleted (*IDH1*/2 mutation in combination with presence of a co-deletion of the entire 1p and 19q chromosomal arms), (2) diffuse astrocytoma, *IDH* mutant; (*IDH1*/*2* mutation without 1p19q co-deletion), and (3) diffuse astrocytoma, *IDH1*/*2* wildtype. Other frequently reported genetic changes in glioma are *CIC, FUBP1, TP53, ATRX, TERT* promoter mutations and copy number changes of chromosome 7, 9, and 10 [1, 2, 4, 5]. Although the 2016 CNS WHO update is robust and provides a more accurate prognosis estimation, there is still variation in outcome within the different entities and the grading of glioma is still depending on histological features. Prognostication might be further improved if additional molecular markers can be identified that correlate with prognosis. These markers may facilitate treatment decisions, in particular for early radiotherapy and adjuvant chemotherapy versus a wait and watch policy after surgery. This is in particular relevant, as the currently used criteria (age of 40, less than gross total resection) are quite arbitrary, and in some patients that are 40 years or older with a less than gross total resection, a wait and watch period of years is possible [6–8]. Thus, molecular factors showing a clinically significant relation with outcome would be highly welcome.

Several studies have tried to further stratify molecularly defined glioma, most of them in anaplastic glioma. For example, some studies showed that loss of chromosome 9p or specifically the 9p21.3 region is associated with a worse prognosis in various subtypes of grade III and IV glioma [9–11]. This could however not be validated in a recently published large cohort of grade II and III glioma [12]. In the present study we aimed to identify molecular prognostic markers for grade II *IDH* mutated astrocytoma (in particular loss of 10q, trisomy of chromosome 7, *PTEN* mutations) and in grade II *IDH* mutated 1p19q-codeleted oligodendroglioma (in particular loss of 9p21.3, *CIC, FUBP1*, and *PTEN* mutations). To our knowledge there are no published studies that investigated prognostic impact of these markers specifically in molecularly defined grade II glioma. Therefore, in a well-defined cohort of histologically proven supratentorial adult grade II glioma we used a targeted Next-Generation Sequencing panel for molecular classification and evaluated the prognostic value of glioma specific molecular markers.

## Methods

### Patient selection

For this study we used a cohort of patients from a project on extent of resection in grade II glioma [13]. Adult patients (age ≥ 18 years) with histopathologically confirmed supratentorial grade II glioma were included. Tissue samples were collected in two Dutch hospitals (Erasmus MC Cancer Institute, Rotterdam; and Elisabeth-TweeSteden Hospital, Tilburg). Histopathological diagnosis and low grade was confirmed by a dedicated neuropathologist (J.M.K.). Time-window of patient inclusion was 2003–2016. For clinical factors, age, KPS, type of surgery, and treatment after surgery were collected. This study was approved by the medical ethics committee of Erasmus MC.

### DNA extraction

DNA was isolated from formalin-fixed-paraffin-embedded (FFPE) tissue blocks. Tissue areas with high percentage of neoplastic cells (preferably > 70%, but at least 50%) were manually macrodissected from 10 µm sections. Macrodissected tissue was digested using Proteinase K incubation at 56 °C overnight in presence of 5% Chelex 100 resin (Bio-Rad). After overnight incubation, proteinase K was inactivated at 90 °C for 10 min. Next, dissolved DNA was separated from Chelex resin and cell debris by centrifugation at 20 g for 5 min. DNA concentration was measured using the Qubit 3.0 Fluorometer according to manufacturer’s protocol (Life Technologies).

### Next-Generation-Sequencing

We used Next-Generation Sequencing to assess mutational and copy number status of selected genes and chromosomes. The primer panel consisted of primers for glioma specific genes of interest (hot spot regions, or whole gene) and primers for highly polymorphic single nucleotide polymorphisms to detect large genomic alterations in chromosomes of interest. Chromosomal imbalances and loss of heterozygosity (LOH) were estimated as described previously [3]. An overview of the targeted hotspots/whole genes and chromosomes is shown in Supplementary Table 1. Sequencing was performed with the Ion Torrent Personal Genome Machine or Ion S5 (Life Technologies). *TERT* promoter mutational status (C228T and C250T mutation) was assessed in a separate assay as described before (SNaPshot, Life Technologies) [3].

### Survival

All patients were followed until death or censored at date of last follow-up. Overall survival (OS) was measured from the date of diagnostic scan until date of death or censorship. Date of death was provided by patient records or the Municipal Personal Records Database. The database was developed and maintained at Erasmus MC, and locked on March 21st 2018.

### Statistical analysis

All analyses were performed using R (3.3.2) and RStudio (1.0.44). Overall survival was measured as time between date of diagnostic scan and date of death or censorship. Overall survival is shown in Kaplan–Meier plots (ggplot2 package in R). Univariable analyses were performed using the Log-rank test and multivariable analyses with Cox proportional-hazards models. Categorical data were analyzed with Pearson’s Chi square test or Fisher’s exact test when assumptions of the Chi square test were violated (as indicated in the respective tables). Kruskal–Wallis test was used for continuous data. All calculations were two-sided tests, with a P value < 0.05 considered as statistically significant.

## Results

We identified 246 patients with a pathologically confirmed grade II glioma with available FFPE material. Of these, 2 patients were excluded due to insufficient DNA yield and no remaining tissue for DNA isolation, and 14 were excluded from analysis due to sequencing failure (very low coverage and/or uniformity for most amplicons after two attempts). At the time of analysis, 53 patients were reported dead, 15 of the 95 IDH mutated 1p/19q-codeleted oligodendroglioma patients and 38 of the 112 IDH mutated astrocytoma patients.

### Molecular classification

**Fig. 1:**
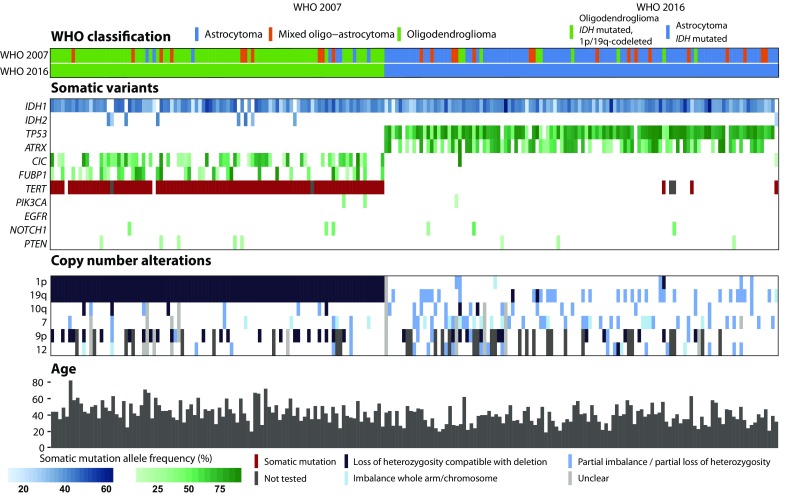
Oncoprint plot with overview of somatic alterations per patient. At the top of the figure the WHO 2007 and WHO 2016 classification are shown. In the middle part all somatic variants and copy number alterations are shown. Patients are separated based on the WHO 2016 classification. *IDH* mutated 1p/19q-codeleted patients are depicted in the left part of the figure and *IDH* mutated astrocytoma patients on the right part of the figure. The bottom part of the figure shows the clinical characteristic age per patient

As we were interested in additional markers for *IDH* mutated grade II glioma, 23 patient samples that were classified as *IDH* wildtype were excluded from further analyses. 207 patients were included in final analyses with a median follow-up of 6.9 years (range 0.4–21.7 years). Clinical characteristics are shown in Table 1. An oncoprint plot with all mutations and copy number alterations for the 207 patients is shown in Fig. 1. Mutation frequencies per molecular subgroup are shown in Table 2. Median overall survival per CNS WHO 2016 molecular subgroup was consistent with literature (Supplementary Fig. 1).

**Table 1:** Patient characteristics

Characteristics	Oligodendroglioma		Astrocytoma IDHmt		P
	N	%	N	%	
Patients (n)	95		112		
Sex					0.149
Male	49	51.6	71	63.4	
Female	46	48.4	41	36.6	
Age					< 0.0001
Median (IQR)	45	(37–52)	37	(29–45)	
Type of 1st surgery					0.002^†^
Awake craniotomy	51	53.7	54	48.2	
Normal resection	23	24.2	49	43.8	
Open biopsy	7	7.4	2	1.8	
Stereotactic biopsy	14	14.7	7	6.2	
Preoperative KPS					0.064
Median (IQR)	100	(100–100)	100	(90–100)	
Histopathological diagnosis					
Grade II astrocytoma	8	8.4	87	77.7	
Grade II oligodendroglioma	77	81.1	9	8.0	
Grade II oligo-astrocytoma	10	10.5	16	14.3	
Treatment after 1st surgery					< 0.0001
Wait and scan	52	54.7	52	46.4	
Chemotherapy	24	25.3	5	4.5	
Radiotherapy	16	16.8	42	37.5	
Chemoradiation	3	3.2	13	11.6	
Follow-up (years)					
Median (range)	8.0	(0.9–21.7)	6.1	(0.4–16.7)	

^†^Fisher’s exact test

**Table 2:** Frequencies of gene mutations per WHO subgroup

Gene	IDH mutated astrocytoma		IDH mutated 1p/19q-codeleted oligodendroglioma	
	N	%	N	%
*IDH1*	111	99.1	87	91.6
*IDH2*	1	0.9	8	8.4
*TP53*	105	93.8	0	0
*ATRX*	75	67	0	0
*CIC*	2	1.8	52	54.7
*FUBP1*	0	0	36	37.9
*TERT*	3	2.7	91	95.8
*PIK3CA*	1	0.9	2	2.1
*EGFR*	0	0	0	0
*NOTCH1*	3	2.7	3	3.2
*PTEN*	3	2.7	5	5.3

### IDH mutated astrocytoma

**Fig. 2:**
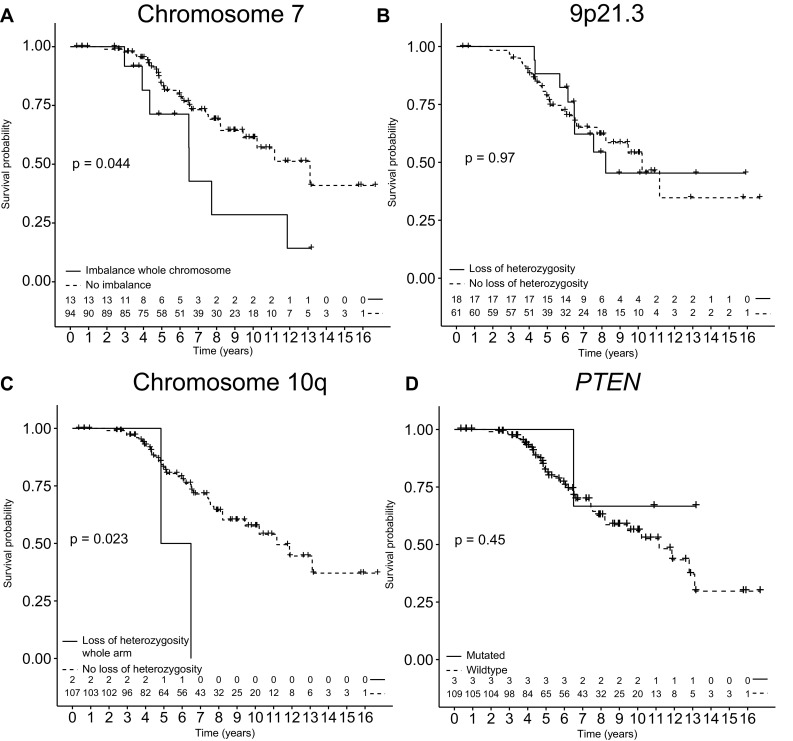
Kaplan–Meier plots with overall survival of *IDH* mutated astrocytoma patients. Kaplan–Meier plots with overall survival of *IDH* mutated astrocytoma patients stratified for presence of **a** imbalance pattern consistent with trisomy of chromosome 7, **b** loss of 9p21.3 region, **c** loss of chromosomal arm 10q, **d** and presence of a *PTEN* mutation

107 of the 112 *IDH* mutated astrocytomas could be reliably evaluated for imbalance of chromosome 7: 13 samples showed imbalance compatible with trisomy of the entire chromosome 7. In univariable analysis trisomy of chromosome 7 was significantly associated with shorter overall survival (Log rank P = 0.044). However, this survival difference lost significance when correcting for age and KPS (HR 2.22; 95% CI 0.95–5.20; P = 0.066). Only two samples showed loss of entire chromosomal arm 10q, both with a relatively poor overall survival of less than 8 years (Fig. 2). Loss of 9p21.3 (n = 18) was not associated with outcome. Out of the 18 patients with loss of 9p21.3, 13 showed loss of the entire 9p chromosomal arm, which was also not associated with outcome. A *PTEN* mutation was detected in 3 patients (all three no loss of 10q) and did not show an impact on outcome (Fig. 2).

### IDH mutated 1p/19q-codeleted oligodendroglioma

**Fig. 3:**
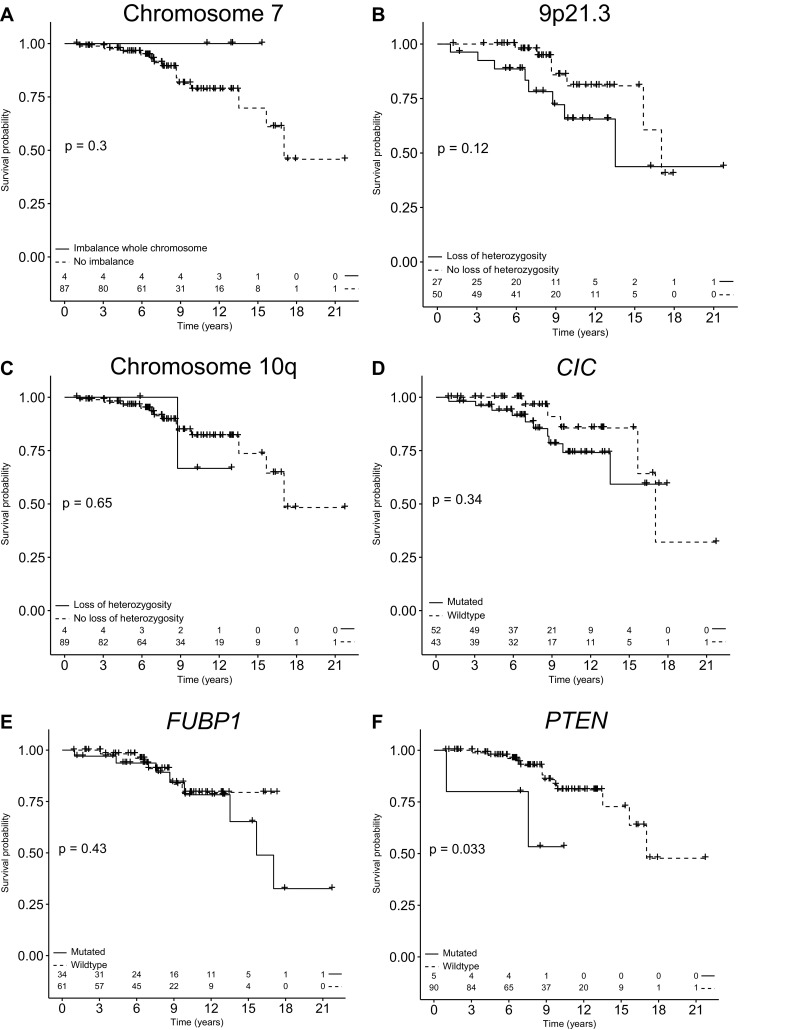
Kaplan–Meier plots with overall survival of *IDH* mutated 1p/19q-codeleted oligodendroglioma patients. Kaplan–Meier plots with overall survival of *IDH* mutated 1p/19q-codeleted oligodendroglioma patients stratified for presence of **a** Imbalance pattern consistent with trisomy of chromosome 7, **b** loss of 9p21.3 region, **c** loss of chromosomal arm 10q, **d** presence of *CIC* mutation, **e** presence of *FUBP1* mutation, **f** and presence of a *PTEN* mutation

In *IDH* mutated 1p/19q-codeleted oligodendroglioma, both *CIC* and *FUBP1* mutations were frequent events, but neither was associated with prognosis (Fig. 3). In 27 out of the 77 patients that could be reliably evaluated for copy number changes on chromosome 9p, loss of 9p21.3 was found. Of these, 23 showed loss of entire chromosomal arm 9p. No homozygous deletions or mutations of *CDKN2A* were detected. Loss of 9p21.3 did not have significant impact on overall survival (Log rank P = 0.12) (Fig. 3). Additional analysis for impact of loss of entire 9p did not show different results. Trisomy of chromosome 7 and loss of chromosomal arm 10q were present in a few samples, both without impact on overall survival (Fig. 3). Five patients were *PTEN* mutated and showed a significantly shorter OS in univariable analysis (Log rank P = 0.033). This survival difference was not significant anymore when correcting for age and KPS (HR 3.73; 95% CI 0.78–17.76; P = 0.097).

### Exploratory analyses

On further exploratory analyses, we found no other molecular markers that significantly impact on overall survival in molecularly defined LGG with the targeted sequencing panel we used.

### Validation of findings in the TCGA

We aimed to validate our findings in the publically available dataset *The Cancer Genome Atlas* (TCGA). In the TCGA dataset, 9 out of 72 grade II IDH mutated astrocytoma showed trisomy of chromosome 7, but this was not significantly associated with a difference in overall survival in univariate analysis (Log rank P = 0.3). We also analyzed the TCGA dataset for presence of loss of 10q in grade II *IDH* mutated astrocytoma. Only one sample showed loss of 10q, with a poor survival (1.75 years). Among the 47 grade II *IDH* mutated 1p/19q-codeleted oligodendroglioma samples in the TCGA dataset, there were no samples with a *PTEN* mutation, confirming the rarity of this event.

## Discussion

In this study we aimed to investigate the prognostic impact of additional molecular markers in grade II *IDH* mutated astrocytoma and grade II *IDH* mutated 1p/19q-codeleted oligodendroglioma. We used a targeted NGS panel to investigate genes that are frequently mutated in glioma and to investigate regions that are frequently reported to have copy number changes.

Loss of chromosome 9p or in particular the 9p21.3 region is a frequently reported copy number variation in all glioma subtypes [2, 9–11]. Several studies investigated the impact of loss of 9p/9p21.3 on prognosis. A meta-analysis published in 2015 pooled the data of 13 different studies that were published between 2002 and 2013 [10]. Although most individual studies in this meta-analysis did not show a survival difference, the pooled data showed that loss of 9p was significantly associated with a poorer prognosis. However, most studies in this analysis did not take into account confounding factors such as *IDH1*/*2* mutational status, and a subgroup analysis of types of gliomas showed that impact of loss of 9p on overall survival was particularly present in the glioblastoma subtype. A recent study specifically focused on the impact of 9p loss in grade II and III glioma and found that loss of 9p is an independent prognostic factor in IDH mutated glioma, however, the effect was most clear in *IDH* mutated astrocytomas [11]. In opposite, Alentorn et al. found loss of 9p to be a poor prognostic marker in anaplastic oligodendroglioma [9]. A later study by Aoki et al. did not confirm this finding however [12]. Our study is the first that specifically focusses on the impact of loss of 9p21.3 and entire 9p in histologically defined grade II glioma. We could not confirm impact of loss of 9p21.3 region or entire 9p on prognosis in *IDH* mutated astrocytoma nor in *IDH* mutated 1p/19q-codeleted oligodendroglioma. However, a trend towards shorter overall survival in *IDH* mutated 1p/19q-codeleted oligodendroglioma with loss of 9p21.3 is visible, and longer follow-up and larger sample size is necessary for final conclusions. Exact comparison of our data with previous literature is difficult, because of different selection criteria in the different cohorts. Therefore, it is yet unclear if in grade II *IDH* mutated glioma loss of 9p21.3 region can be used as marker of anaplasia or to guide more aggressive treatment strategies.

Trisomy of chromosome 7 is also frequently reported in glioma [2, 14, 15]. In combination with loss of 10q it is considered an early event in glioblastoma *IDH* wildtype and is correlated with dismal prognosis in grade II and III *IDH* wildtype glioma [2, 15–17]. Trisomy of chromosome 7 is also described in lower grade glioma, though less frequently. The impact of trisomy of 7 on overall survival in *IDH* mutated low grade glioma is not clear. To our knowledge no large series are published. Wessels et al. reported that polysomy of 7 was associated with a poorer prognosis in grade II astrocytoma, but this report antedates the discovery of the role IDH mutations in glioma [14]. In our cohort we found that trisomy of chromosome 7 might be a marker of poor prognosis in *IDH* mutated astrocytoma. However, this could not be validated in multivariate analysis nor in the TCGA data, and this observation requires validation in a larger independent cohort to define the clinical value.

*CIC* and *FUBP1* mutations are frequently mutated in *IDH* mutated 1p/19q-codeleted oligodendroglioma and the prognostic impact has been investigated in several series. One study by Gleize et al. reported that inactivating *CIC* mutations in *IDH* mutated glioma correlate with poorer outcome. In other cohorts this effect was not observed [3, 18–20]. In our cohort we also did not find a correlation between *CIC* nor *FUBP1* mutation and prognosis.

Our study has several limitations. The retrospective nature comes with the risk of a selection bias. We tried to avoid selection bias by analyzing a consecutive cohort of all grade II gliomas undergoing surgery within a specified period. Also due to the retrospective nature, treatment was heterogeneous. Furthermore, we may have missed smaller region copy number alterations and other mutations in regions not covered by our NGS panel. We used a diagnostically validated [21] and targeted NGS panel that consists of highly polymorphic SNPs that cover whole chromosomes of interest with roughly 1 SNP per 3 MB. Large scale copy number variations of whole chromosomes or large parts of chromosomes can therefore be reliably detected, but small region or subclonal copy number variations are potentially missed. However, the aim of this study was to validate the impact of large region copy number variations which were described before and that are considered to be early events, and these can be reliably detected with the panel used [21]. Also, it is known that lengthy follow-up in studies on low-grade glioma is necessary for definitive conclusions, so longer of follow up of this dataset is necessary.

In conclusion, in univariable analysis we found a significant shorter overall survival in *IDH* mutated astrocytoma patients with trisomy of chromosome 7, and in *IDH* mutated 1p/19q-codeleted oligodendroglioma patients with a *PTEN* mutation. However, we could not confirm these findings in multivariate analysis or in the TCGA validation set and therefore these findings require validation in other larger series. We could not confirm the impact on OS of LOH of 9p21.3 (the CDKN2A region) which is frequently reported as a progression marker particularly in higher grade glioma. However, we need lengthy follow-up for definitive conclusions. Also, other strategies should be pursued to identify prognostic relevant molecular markers within these IDH mutant glioma subgroups, like methylation patterns and total number of chromosomal aberrations.

## Electronic supplementary material

Below is the link to the electronic supplementary material.

Supplementary material 1 (PDF 91 KB)
