# Supplementary material for: Prognostic relevance of mutations and copy number alterations assessed with targeted next generation sequencing in IDH mutant grade II glioma
Source: J Neurooncol. 2018 Apr 16;139(2):349–57. doi: 10.1007/s11060-018-2867-8 (PMC6096891; doi:10.1007/s11060-018-2867-8)
Supplement: Supplementary file 1 — Supplementary material 1 (PDF 91 KB) [file 11060_2018_2867_MOESM1_ESM.pdf]

Supplementary table 1

overview of targeted genes and chromosomes

Whole gene	Hotspots	SNPs	SNaPshot
TP53	*EGFR* (exon 3+15)	chr1	TERT promoter
FUBP1	*H3F3A* (exon 2)	chr10 (including *PTEN*)	
PTEN	*IDH2* (exon 4)	chr12 (including *MDM2*)	
CIC	*IDH1* (exon 4)	chr19	
CDKN2A	*PIK3CA* (exon 10 + 21)	chr7 (including *EGFR* and *MET*)	
NOTCH1	*BRAF* (exon 11+15)	chr9 (including *CDKN2A*)	
ATRX		chrY	

Supplementary figure 1

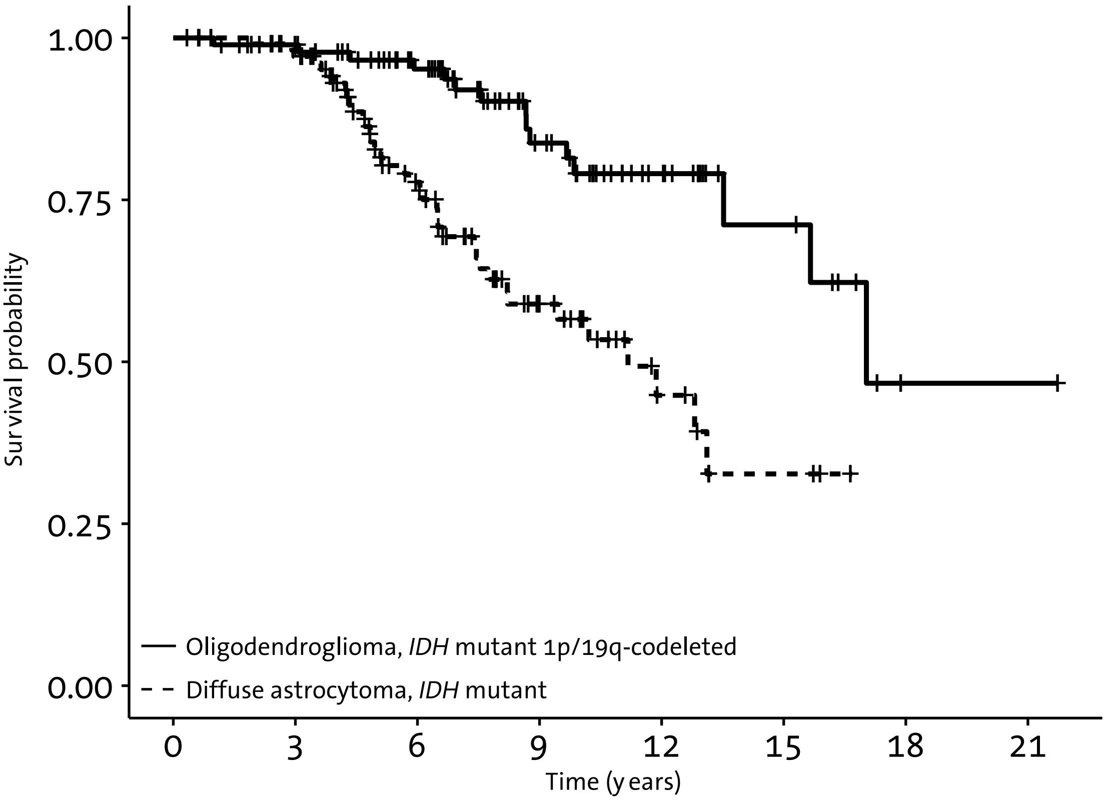

Number at risk

—	95	88	69	38	20	9	1	1
- -	112	101	59	27	9	3	0	0

Kaplan-Meier plot with overall survival stratified by WHO 2016 molecular subgroup. IDH mutated 1p/19q-codeleted patients have significantly longer overall survival (median OS 17.0 years) compared to IDH mutated astrocytoma patients (median OS 11.2 years) (Log rank test: $P < 0.0001$).
